# Magnetic Resonance Spectroscopic Findings of Chronic Lesions in Two Subtypes of Multiple Sclerosis: Primary Progressive Versus Relapsing Remitting

**DOI:** 10.5812/iranjradiol.11336

**Published:** 2013-08-30

**Authors:** Nasrin Rahimian, Hamidreza Saligheh Rad, Kavous Firouznia, Seyed Amir Ebrahimzadeh, Alipasha Meysamie, Hamideh Vafaiean, Mohammad Hossein Harirchian

**Affiliations:** 1 Iranian Center of Neurological Research, Tehran University of Medical Sciences, Tehran, Iran; 2 Quantitative MR Imaging and Spectroscopy Group, Research Center for Cellular and Molecular Imaging, Medical Physics and Biomedical Engineering Department, Tehran University of Medical Sciences, Tehran, Iran; 3 Advanced Diagnostic and Interventional Radiology Research Center (ADIR), Tehran University of Medical Sciences, Tehran, Iran; 4 Department of Radiology, Shohada-e-Tajrish Hospital, Shahid Beheshti University of Medical Sciences, Tehran, Iran; 5 Community Medicine Department, Medical Faculty, Tehran University of Medical Sciences, Tehran, Iran

**Keywords:** Multiple Sclerosis, Chronic Progressive, Multiple Sclerosis, Relapsing-Remitting, Magnetic Resonance Spectroscopy

## Abstract

**Background:**

Multiple sclerosis (MS) is a highly prevalent cause of neurological disability and has different clinical subtypes with potentially different underlying pathologies. Differentiation of primary progressive multiple sclerosis (PPMS) from relapsing remitting multiple sclerosis (RRMS) could be difficult especially in its early phases.

**Objectives:**

We compared brain metabolite concentrations and ratios in patients with PPMS and RRMS by magnetic resonance spectroscopic imaging (MRSI).

**Patients and Methods:**

Thirty patients with definite MS (15 with RRMS and 15 with PPMS) underwent MRSI and their non-enhancing lesion metabolites were measured. N-acetyl aspartate (NAA), Creatine (Cr), Choline (Cho), NAA/Cr and NAA/Cho were measured and compared between the two MS subtypes.

**Results:**

When the two MS groups were compared together, we found that Cr was significantly increased (P value=0.008) and NAA/Cr was significantly decreased (P value=0.03) in non-enhancing lesions in PPMS compared with RRMS. There was no significant difference in NAA, Cho or NAA/Cho between the two MS subtypes.

**Conclusion:**

MRS is a potential way to differentiate PPMS and RRMS.

## 1. Background

Multiple sclerosis (MS) is an inflammatory and demyelinating disease affecting the central nervous system. MS is one of the most prevalent causes of neurological disability especially in the young population (1). The clinical course of the disease can be highly variable. The most common MS subtype affecting approximately 85% of the patients is termed relapsing remitting MS (RRMS) that is characterized by an acute onset of symptoms that remit spontaneously. Another 10-15% of patients experience disease progression from the onset. This is termed primary progressive multiple sclerosis (PPMS) (1, 2).

Magnetic resonance findings have been shown to be helpful in differentiating clinically isolated syndrome (CIS) from definite multiple sclerosis (3), as well as various subtypes of MS, especially PPMS from RRMS. Although the brain appears relatively normal on conventional MRI in PPMS patients, their higher degree of disability suggests more severe pathology and available data suggests that biologically, PPMS differs from RRMS (4, 5). Since application of magnetic resonance spectroscopy (MRS) in patients with MS allows us to acquire information about metabolites within the brain tissue (6-8), it can potentially be applied in the identification of biological changes and the differentiation of MS clinical subtypes (9, 10).

Among the brain metabolites, N-acetyl aspartate (NAA) has the most important signal in the MRS spectra (11). It is a marker of neuronal and axonal metabolism and a decreased NAA concentration suggests loss of neuronal/axonal integrity (11, 12). The Choline (Cho) signal is a specific marker of membrane turnover and the increased Creatine (Cr) can be attributable to gliosis (13-15).

## 2. Objectives

The goal of this study was to compare amounts of NAA, Cr, Cho and their ratios including NAA/Cr and Cho/Cr, in non-enhancing periventricular lesions in PPMS with RRMS patients. We aimed to determine whether there were different patterns of metabolite changes between these MS subtypes. To the best of our knowledge, a scrupulous comparison of NAA, Cho, Cr and their ratios in non-enhancing lesions in PPMS with RRMS using a 3-Tesla scanner has not been reported.

## 3. Patients and Methods

### 3.1. Patients

Thirty patients (15 RRMS and 15 PPMS) with clinically definite MS according to McDonald’s criteria (16) were recruited. Patients who had received corticosteroids within the previous 3 months were excluded because of its potential effect on the enhancement of acute plaques. All patients underwent a full neurological examination and their expended disability status scale (EDSS) was measured. The joint ethics committee at Tehran University of Medical Sciences approved the study. Prior to image acquisition, all subjects provided written informed consent.

### 3.2. Magnetic Resonance Imaging

All MRI and MRS acquisitions were acquired on a 3-Tesla scanner (Magnetom Tim Trio 3T, Siemens, Erlangen, Germany) with an 8-channel head coil in our tertiary university hospital. Axial and sagittal T2-weighted images (TE/TR=3000/96 ms) were acquired. Contrast T1-weighted images (TE/TE=650/14 ms) were obtained in patients 10 minutes after intravenous administration of paramagnetic contrast at a dose of 0.1 mg/kg (Magnevist, Bayer Schering Pharma, Germany).

MRS was performed using multivoxel chemical-shift imaging (CSI) and employing the point-resolved spectroscopy (PRESS) technique in the periventricular region. MRS acquisition was done at the volume of interest (VOI) with the dimension of 8×8 voxels and the field of view (FOV) with the dimension of 16×16 array of spectra, resulting in the voxel size of 1×1×2 cm^3^. The acquisition parameters were spectral bandwidth of 2000 Hz, number of spectral points of 1024, TR equal to 1000 ms and TE equal to 135 ms. In order to perform absolute quantification and to reform signal phase distortion, a single voxel spectroscopy (SVS) was acquired from an external reference phantom with a known concentration using PRESS technique without water suppression. Prior to acquiring the MRS data, manual shimming was performed to optimize magnetic field homogeneity and calibration. The entire protocol took less than 45 minutes.

### 3.3. Voxel Selection

**Figure 1.**
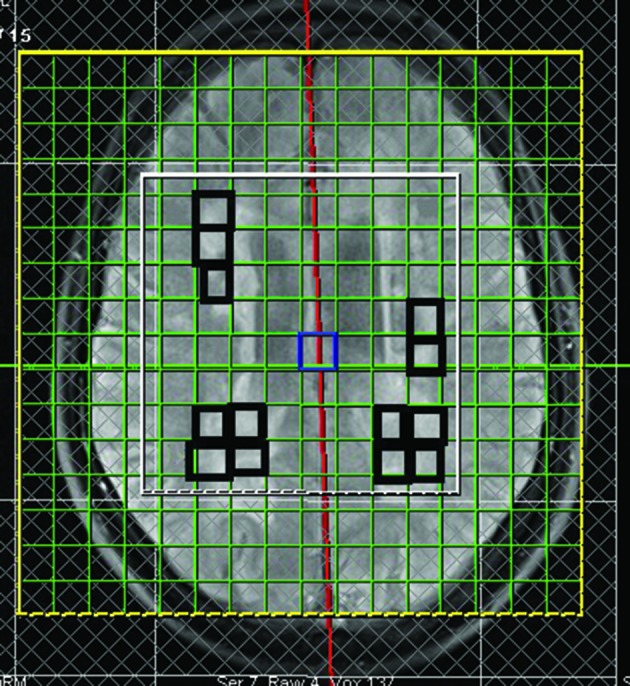
Periventricular lesions marked with black rectangles

A single radiologist, who was blind to the MS subtypes, identified periventricular MS lesions on T2-weighted MRI. Figure 1 shows the area over which spectroscopic imaging was performed with the rectangles on the midsagittal image hyper-intensities. 

To minimize partial cerebrospinal fluid volume effects, we considered voxels that were completely within a lesion, without any apparent involvement of the ventricles or sulci. Only non-enhancing lesions that occupied at least 60% of the voxel were studied in both groups (6). We assessed all non-enhancing legions fulfilling the mentioned inclusion criteria. Overall, there were 94 included lesions for RRMS patients (mean number of plaques±SD: 7.8±3.09) and 83 lesions for PPMS patients (mean number of plaques±SD: 7.5±3.2).

### 3.4. MRS Quantification

Quantification of the spectra was performed in the time domain using subtract-QUEST (17). In subtract-QUEST MRSI, we used an optimized metabolite basis-set to estimate the metabolites as well as the base-line. The simulated basis-set was created in NMR-SCOP of the jMRUI software. Fitted peaks for the metabolites included the range of chemical-shift as follows: NAA (1.9, 2.1), Cr (2.9, 3.1) and Cho (3.1, 3.3) (17). All metabolite concentrations were expressed relative to Cr. We performed external referencing to compute the absolute concentration of metabolites against an external reference phantom containing the known concentration of water (18).

The result of quantification was evaluated using Cramer-Rao bound (CRB) that is a method to describe unreliability of estimation. According to some criteria such as Cramer-Rao minimum variance bound (17), the accurate peak fitting reliability was confirmed.

### 3.5. Statistical Analysis

All statistical analyses were performed using Statistical Package for the Social Sciences (SPSS) for Windows Ver. 17.0 (SPSS Inc., Chicago, Illinois, USA). Statistical differences were evaluated with Student’s T or the Mann-Whitney U tests depending on the normality of data. In addition to comparing the metabolite concentrations and their ratios, we evaluated the differences in important variables (age, disease duration and EDSS) between two MS subtypes. P values of less than 0.05 were considered significant for all analyses.

## 4. Results

### 4.1. Demographics

Demographic and clinical data (EDSS and disease duration) are summarized in Table 1. There was no statistically significant age difference between PPMS and RRMS patients (P=0.16). No significant difference was detected in disease duration between the two groups (P = 0.16). The PPMS group had a significantly higher EDSS than the RRMS group (P=0.003). 

**Table 1.** Demographic and Clinical Data of Participants

	PPMS*	RRMS*
**Number of subjects**	15	15
**Age (y)**	40.9± 9.42	37.4± 7.04
**Disease duration (y)**	5± 2.5	3.71± 2.1
**EDSS**	3.66± 2.49	1.16± 1.58

Abbreviations: EDSS, Expanded Disability Status Scale; PPMS, Primary Progressive Multiple Sclerosis; RRMS, Relapsing Remitting Multiple Sclerosis *Values are expressed as mean ± standard deviation.

### 4.2. Non-Enhancing Lesions

Mean values of NAA/Cr, NAA/Cho, NAA, Cho and Cr and their comparisons for non-enhancing lesions in PPMS and RRMS are summarized in Table 2. Analysis of data showed that NAA/Cr measures for non-enhancing lesions in PPMS patients were significantly lower than the RRMS group (P=0.03). Cr was significantly higher in the chronic lesions of the PPMS group than those of the RRMS group (P=0.008). No statistically significant differences were found in NAA/Cho, Cho or NAA between the two groups. 

**Table 2.** NAA, Cr and Cho Absolute Concentrations (ppm) and Ratios for Non-Enhancing Lesions in Each Multiple Sclerosis Subtype and P Values of Between Group Comparisons

	RRMS-Lesion*	PPMS-Lesion*	P Value
**NAA/Cr**	1.71±1.4	0.91±0.55	0.03
**NAA/Cho**	4.27±1.84	4.38±2.57	0.84
**NAA**	8.45±0.88	7.87±0.7	0.08
**Cho**	1.73±0.35	2±0.5	0.12
**Cr**	5.21±0.75	6±0.57	0.008

Abbreviations: Cr, Creatine; Cho, Choline; NAA, N Acetyl Aspartate; PPMS, Primary Progressive Multiple Sclerosis; RRMS, Relapsing Remitting Multiple Sclerosis *Results are expressed as mean ± standard deviation.

## 5. Discussion

Data from studies evaluating natural history (19), magnetic resonance imaging (20) immunology (21) and histopathology (4) of MS suggest that the PPMS differs significantly from RRMS. Being able to predict a patient’s MS course is highly desirable but lacking. Nowadays, conventional T2-weighted MRI is widely used to assess lesion burden in MS. However, T2 lesion burden has a poor correlation with disability in MS (22, 23) and consequently, it cannot differentiate the subtypes of the disease (23). It is accepted that the MRS quantification may provide evidence of irreversible tissue damage in MS by estimating brain metabolite quantities more specific than T2-weighted MRI (14).

When comparing PPMS patients with RRMS patients we found that:

(a) In agreement with previous studies, there was a significant NAA/Cr decrease in non-enhancing lesions (7) of PPMS patients when compared to RRMS. It could be related to either a more pronounced NAA reduction or Cr increase in PPMS than in the RRMS subtype. This finding is most probably due to different pathophysiological mechanisms such as axonal damage (reduced NAA) and/or gliosis (increased Cr) in PPMS patients. The NAA/Cr ratio reduction may suggest a neuronal/axonal integrity loss in these patients (14).

(b) We found that Cr concentration (a marker of gliosis) was significantly higher in non-enhancing lesions of PPMS than that of RRMS. This increase in Cr reflects a more severe gliosis in our PPMS group and is consistent with other studies (7). Age has a well-established direct correlation with the increased brain Cr level (7) and because the PPMS subtype tends to start in an older age than RRMS, it could be a potential confounding factor for increased Cr level in PPMS. Since the MS groups in our study had no difference in their disease duration and age, the increased Cr concentration in PPMS patients can be attributed to the more severe gliotic nature of the PPMS subtype.

(c) In agreement with Suhy J et al. (7), no significant difference in Cho or NAA/Cho measures was detected between the chronic lesions of PPMS and those of the RRMS patients.

(d) In contrast to some other studies (7, 24), no NAA difference was detected between our MS subtypes. This may be explained by the fact that our MS patients had no significant difference regarding age or disease duration and we only included chronic non-enhancing lesions.

The data from lesions could be contaminated by volume averaging from nearby focal lesions or CSF. To avoid this, we carefully selected voxels that predominantly included the lesion tissue. Nevertheless, contamination from adjacent tissue or partial volume effects cannot be completely prevented because of the coarse MRS spatial resolution.

In conclusion, these results suggest that Cr concentration or NAA/Cr ratio in non-enhancing lesions can potentially differentiate between RRMS and PPMS subtypes. Further studies comparing PPMS and RRMS groups with the same EDSS are suggested.

We also suggest designing a longitudinal study on PPMS and RRMS separately to track the changes of metabolites with disease chronicity and time.
